# Exploring the possibilities and limitations of climate-smart agriculture for SDG 2 in West Africa

**DOI:** 10.3389/fnut.2026.1820766

**Published:** 2026-08-10

**Authors:** Edidiong Samuel Akpabio, Kehinde Oluwaseun Omotoso, Samuel Olawale, Adeola Oluwakemi Ogunbadeniyi

**Affiliations:** 1 Department of Political Social Science, Trinity University, Lagos, Nigeria; 2 Teesside University International Business School, Middlesbrough, United Kingdom; 3 University of South Africa College of Graduate Studies, Pretoria, South Africa; 4 Department of Political Science, Tai Solarin University of Education, Ijebu Ode, Nigeria; 5 Department of Political Science, University of Benin, Benin City, Nigeria

**Keywords:** climate smart agriculture, development, food security, SDGs, West Africa

## Abstract

Climate change continues to exacerbate food insecurity across West Africa, posing a major threat to the achievement of Sustainable Development Goal 2 (SDG 2: Zero Hunger). Despite the multifarious interventions implemented to mitigate its effects, climate vagaries such as floods, erratic weather patterns, droughts, and farmer-herder conflict, as well as weak policy implementation, have coincided with continued population growth, thereby worsening food insecurity in the sub-region. This study examines the possibilities and limitations of engaging climate-smart technologies to achieve SDG 2 in West Africa. The study utilized secondary data sources with the aid of a qualitative methodology grounded in an integrative review, guided by the PRISMA 2020 guidelines. The study narrowed its analysis to 85 relevant studies retrieved from electronic databases such as Scopus, Google Scholar, and EBSCOhost. The entire body of literature on the research was synthesized and consolidated through three theoretical frameworks: Climate Resilience Theory, the Sustainable Food Systems (SFS) Framework, and Agricultural Innovation Diffusion Theory. Findings indicate that while climate-smart agriculture (CSA) recorded a wide range of successes in countries such as Mali, Ghana, Nigeria, Senegal, and Burkina Faso, its adoption rates were notably low in the sub-continent due to the challenges of technological know-how, illiteracy, and weak government policies, among other factors. We concluded that achieving SDG 2 requires a holistic ecosystem approach that integrates digital literacy and climate-smart outputs into regional initiatives such as the WAICSA framework, strengthens regional cooperation, and encourages culturally sensitive technologies to mitigate the sub-region’s vulnerability to persistent food deficit fueled by a worsening climate trajectory.

## Introduction

It remains a worrisome development that food insecurity in Africa is surging despite the multifarious efforts to stem its tide (1). This is in sharp contrast to the Caribbean and Latin American regions (2). Although food insecurity is a global occurrence, as observed by Jin et al. (3), who report a staggering estimate of approximately 278 million people affected by food insecurity in the world. This figure, apart from being high, poses significant threats to the other 16 sustainable development goals (SDGs) while making the 2050 food security goal for the continent appear like a mere pipe dream. With the concept of sustainable development, which is drawn from the field of ecology, becoming a defining feature of our everyday life, there is a need to ensure it is expeditiously attained (3). Preliminary studies have shown that climate variability, economic challenges, and a myriad of other factors continually stifle West Africa’s prospects for achieving SDG 2 (4). Cabot (5) bemoans the situation, as he posits that conflicts between farmers and herders have equally not helped matters, as the sub-region continually battles issues of food insecurity, which are exacerbated by these altercations.

Attaining food security in the West African sub-region remains a herculean task due to a myriad of factors. Jimoh et al. (6), who argue that West Africa’s performance on SDG 2 is dismal, corroborate this view. Furthermore, Ae-Ngibise et al. (7) and Molokwa and Khazamula (8) argue that some West African states are battling famine and acute food shortages due to colonial and post-colonial factors, with recent floods further exacerbating food insecurity in the region (9). This sordid state of affairs (food insecurity) is often fueled by factors such as widespread poverty, which has further exacerbated the challenge in the sub-region (4, 10). Climate dysfunctions and the numerous strategies deployed to defeat them are rooted in fundamental security. Khan et al. (11) capture it more succinctly by positing that climate change has exacerbated food security challenges in the international system (116). While this is a global occurrence (12), studies have shown that the West African sub-region seems to bear the brunt more. Explosive population growth in the sub-continent, for instance, remains a major challenge to SDG 2 (13). Although it has been acknowledged that the Economic Community of West Africa, which is the sub-regional coordinating body of the West African continent, has played a very significant role in promoting the expedited attainment of the global goals (14), SDG 2 attainment seems to be far-fetched, given the myriad of problems confronting this particular goal in the face. This gloomy state of affairs in the region, despite its potential, does not sit well with Juju et al. (15), who expressed concerns about the inability of most West African states to realize their full potential in food security. In an era of sustainability, as evident in the global goals, smart technologies have been infused into diverse areas of human engagement to drive development. To exemplify further, in sectors such as energy generation, healthcare, and agriculture, among others, technologies now play a crucial role (16). This explains the introduction of climate-smart agriculture as a remedy to address the menace of climate change and enhance food security in the sub-region. Towing the same line of thought, Liu et al. (17) affirm that the transformation of agricultural production from small-scale to large-scale operations has made the use of climate-smart agriculture highly expedient.

While considerable success has been achieved in food security through Climate-Smart Agriculture, the legion of problems that continue to plague this new strand of agriculture in the West African sub-region are plenteous. Taking into consideration the aforementioned factors, the crux of this study is to holistically examine the limitations that have adversely impacted this agricultural approach’s ability to deliver optimal outcomes in the sub-region, while unearthing the possibilities that could be harnessed from it. This study builds on the study by Partey et al. (18), who interrogated the prospects of climate-smart agriculture on the continent, by extending the analysis to examine its relationship with SDG 2 within the West African sub-region. A unique contribution made by this study to the body of literature is its assessment of climate-smart agriculture and food security across the entire West African sub-region, which, to the best of our knowledge, has not been done by any researcher identified in our literature search. This study has, therefore, become particularly relevant because of the gap it seeks to fill, given that preliminary research indicates that adopting enhanced agricultural practices is key to promoting food security (19). This forms the foundation of this study, as we posit that adoption of climate-smart agriculture for the attainment of SDG 2 is akin to a double-edged sword, presenting both opportunities and limitations. This study, therefore, stands out from existing studies due to the sub-continental approach it adopts by focusing on West Africa, unlike other studies that focus on individual countries.

## Methodological description

Research methods, beyond providing a blueprint for research to thrive, also provide a framework for scientific inquiry. It is on this basis that the study employs a qualitative research method, grounded in an integrative literature review. Russell (20), in his evaluation of the integrative review, opined that by adopting this framework, researchers can identify gaps and seek to fill them, while also expanding the frontiers of knowledge can be expanded (21). Shuck (22) further supports this research approach, positing that the integrative literature review helps researchers harvest and synthesize data from a myriad of sources. This approach, in addition to enriching research activities, also helps scholars make use of current literature. Furthermore, this study systematically adopted the Preferred Reporting Items for Systematic Reviews and Meta-Analyses (PRISMA 2020) guidelines, which guided the search strategy, screening criteria, and selection parameters.

We relied on secondary literature from book chapters, journal articles, policy briefs, and conference proceedings, extracted from three electronic databases, including Scopus, Google Scholar, and EBSCOhost, to strengthen the subject of discourse on climate-smart agriculture (CSA) and SDG 2 (Zero Hunger) in West Africa. Core thematic keywords were combined through specific Boolean operators using search strings such as (“Climate-Smart Agriculture” OR “CSA” OR “climate-smart practices”) AND (“SDG 2” OR “Zero Hunger” OR “food security”) AND (“West Africa” OR “ECOWAS” OR “Nigeria” OR “Ghana” OR “Burkina Faso” OR “Mali” OR “Senegal”).

We applied the inclusion and exclusion criteria to determine study eligibility. Critically, parameters solidifying the principle of inclusion include literature, books, chapters, and conference proceedings that comprehensively explain CSA technologies and their impact on achieving SDG2 metrics, such as food availability, access, utilization, and stability, within West Africa. Moreover, the exclusion criteria systematically expunged literature, including non-scholarly blogs, studies published prior to 2021, and research conducted outside the Western African region. However, cases in which articles, though beyond the itemized time frame, were included due to their relevance to the topic under discussion.

To systematically execute the search strategy, the four phases of the PRISMA framework were implemented through a multi-stage screening process. The initial database queries and manual searches yielded a pool of 445 records, comprising 150 from Scopus, 195 from Google Scholar, 75 from EBSCOhost, and 25 from gray literature sources. In the process of screening, reference management software was used to remove 115 duplicate entries, leaving 330 unique records for preliminary evaluation. The titles and abstracts of these 330 records were screened against the inclusion criteria, resulting in the exclusion of 190 records that did not align with the core subject matter, while we identified 140 reports for full-text retrieval.

From 140 reports remaining which were retrieved successfully they were subjected to the rigorous eligibility assessment shown in the exclusion of 55 reports for documented justifications; 22 papers were excluded because they are published before 2021; 18 papers presented geographic mismatch which focus on Sub-Saharan Africa lacking provision of generalized data for West African sub-region, with 15 papers lacking explicit discourse on SDG 2, as they purely give an environmental analysis on climate adaptation with no direct metrics on agricultural output for food security. Based on the foregoing, a total of 85 unique citations met the inclusion criteria, as they provided accurate analytical information on CSA for achieving SDG 2 in West Africa (see Tables 1, 2).

**Table 1:** Distribution of included studies by publication year and source type (*N* = 85).

Year range	Journal articles	Book chapters/Books	Policy/Reports	Conference papers	Total
2021–2022	12	4	3	2	21
2023–2024	15	5	4	1	25
2025–2026	28	6	4	1	39
Total	55	15	11	4	85

**Table 2:** Thematic classification of reviewed studies on climate-smart agriculture and SDG 2 in West Africa (*N* = 85).

Theme	Number of studies	Key references
CSA Practices and Adoption Barriers	26	Ogunleye et al. (10), Liu et al. (17), Partey et al. (18), Akpabio et al. (24), Varela et al. (27), Wudil et al. (31), Seran et al. (33), Akinsemolu et al. (37), Chaurasia et al. (38), Mangole et al. (39), Asenso Barnieh et al. (40), Morchid et al. (44), Sauer et al. (45), Adepoju et al. (47), Akpabio et al. (50), Sorgho et al. (51), Ariom et al. (69), Bayala et al. (70), Ewulo et al. (71), Agyekum et al. (76), Akpabio et al. (77), Tambol et al. (78), Paul et al. (79), Zougmoré et al. (82), Mutengwa et al. (84)
Impacts on Food Security (SDG 2)	23	Otekunrin (1), Ae-Ngibise et al. (7), Enworo (9), Chadare et al. (13), Akpabio et al. (24), Atukunda et al. (25), Omokpariola et al. (26), Saidu (29), van Dijk and Djindil (30), Kurebwa and Kurebwa (32), Charles et al. (46), Adepoju et al. (47), Oluwole and Olagunju-Yusuf (48), Akpabio et al. (50), Food and Agriculture Organization of the United Nations, International Fund for Agricultural Development, United Nations Children’s Fund, World Food Programme, and World Health Organization (52), Food Security Information Network and Global Network Against Food Crises (53), Modise (68), Paul et al. (79), Osabohien et al. (81), Bouët et al. (86)
Policy, Governance, and Regional Initiatives	14	Jimoh et al. (6), Juju et al. (15), Azrour et al. (16), Enwa et al. (43), African Development Bank (61), ISF Advisors (62), International Food Policy Research Institute (63), Westcott (65), ECOWAS Commission (66), Regional Agency for Agriculture and Food (64), Vanguard News (72), African Union (73), Bouët et al. (86)
Climate Change, Conflicts, and Vulnerability	12	Anser et al. (4), Cabot (5), Enworo (9), Akpabio et al. (36), Institute for Security Studies (54), Office for the Coordination of Humanitarian Affairs (55), Efobi et al. (56), Cadre Harmonisé (57), World Food Programme (58), World Food Programme (59), Asamaan (83)
Digital and ICT innovations in CSA	7	Akpabio et al. (24), Chaurasia et al. (38), Mangole et al. (39), Zougmoré and Partey (42), Enwa et al. (43), Morchid et al. (44), Akpabio et al. (50), Nabi et al. (75)
Gender, social inclusion, and nutrition	3	Amoak et al. (41), Zougmoré and Partey (42), Osabohien et al. (81)

Source: Author’s compilation.

### Conceptual and theoretical discourses: climate-smart agriculture and food insecurity in West Africa and beyond

Climate-smart agriculture (CSA) has emerged in scholarly literature as a multi-layered governance framework designed to address the intersection between environmental volatility and agricultural productivity. Scholars such as (98, 100, 105, 108, 113) have reached a consensus in defining climate-smart agriculture (CSA) as a unified governance framework designed to innovate and disseminate technology- and data-driven agricultural methods that enhance crop productivity by building resilience to climate change, reducing greenhouse gas emissions, and managing disaster risk. Wang et al. (23) equally lent their voice to the need to improve crop resilience with the aid of robust strategies such as CSA. Operationally, the core tenets of CSA require (a) the necessity of finding site-specific solutions to attain food security in the face of climate change; and (b) strengthening social and production systems’ resilience as well as broad-based approaches to risk management, both in ex-ante and ex-post.

Despite this conceptual clarity, translating these principles into functional practice remains a herculean task within the Global South, particularly across West Africa. This sub-region has been bedeviled by food insecurity, a crisis that threatens to trigger deep societal fractures if steps are not taken to nip it in the bud (24). The primary drivers of this menace are the effects of climate change anomalies, which adversely affect agricultural productivity and have wider implications for the timely actualization of the United Nations Sustainable Development Goal 2 (Zero Hunger). Expanding the analytical scope to the continental level reveals that food insecurity in the region has continually intensified due to compounding pressures from a complex array of environmental and structural stressors (25, 26).

To move beyond a descriptive account of these regional vulnerabilities, this discourse utilizes a unified conceptual matrix comprising Climate Resilience Theory, the Sustainable Food Systems (SFS) Framework, and the Agricultural Innovation Diffusion Theory to evaluate the interface between CSA and food security. Climate Resilience Theory expounds how smallholder agricultural structures respond to environmental anomalies by distinguishing between the capacity to recover from a temporary shock (absorptive capacity) and adaptive capacity, which involves long-term structural modifications to manage future systemic risks. Evidence from West Africa shows that the current farming methods remain in some form of low-level absorption, where adverse weather events—such as extreme weather, severe drought, or fluctuating precipitation—are directly responsible for increasing food insecurity, rather than causing systematic change (27, 87, 92).

Targeted interventions, such as participatory *vulnerability mapping and farmer field schools, which aim to tailor to local soils, rainfall patterns, and staple crops such as maize, millet, sorghum, groundnut, and rice*, are a transition agent from a vulnerable production system in West Africa to sustainable adaptive capacity. Methodologically, this adaptation includes conservation *agriculture* practices focused on minimal *ploughing to preserve soil structure and* on post-harvest residue retention to conserve soil moisture. These methods are frequently paired with manual direct-seeding implements (e.g., hoes or jab planters), localized slash-and-mulch systems, or animal-drawn rippers designed for in-field rainwater harvesting (89, 94, 99). Furthermore, integrating these approaches into agroforestry systems through the incorporation of nitrogen-fixing trees such as *Faidherbia albida* or *Gliricidia sepium* along field boundaries, alongside soil fertility nutrient replenishment (applying 5–10 tonnes of on-farm manure per hectare) and micro-water management structures (contour bunds, trenches, and small-scale retention reservoirs) establishes a robust ecological defense mechanism against weather fluctuation (102, 112). In terms of cost, studies by (89, 101) assert that the adoption cost for a smallholder farmer ranges between US$200–450 per hectare, covering initial investments in improved seeds, basic tools such as hoes or jab planters, and initial labor. However, while low-cost practices such as legume rotation may cost under US$100 per hectare, high-intensity technologies, such as drip irrigation, may exceed US$200 per hectare, which may be costly for resource-poor households, but such costs can be offset through subsidies, microcredit, group purchasing via cooperatives, or government programs under frameworks such as ECOWAP and CAADP (89, 101).

**Figure 1:**
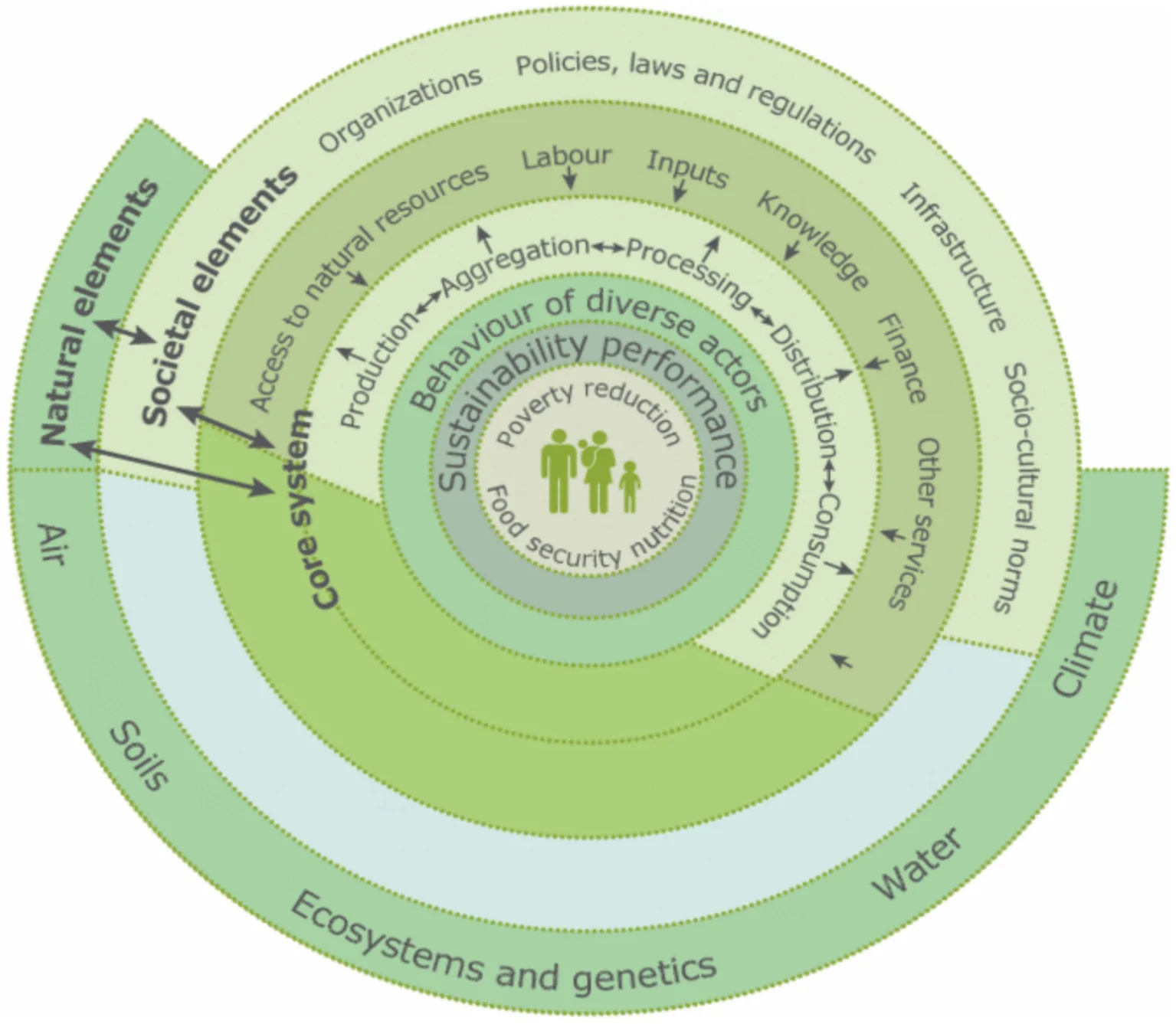
Sustainable food systems framework matrix. Adapted from the FAO (28), licensed under CC BY-NC-SA 3.0 IGO.

Furthermore, because regional food security is not only a matter of agro-ecological output, the Sustainable Food Systems (SFS) Framework expands the analytical framework by viewing food security as the outcome of the interconnectivity between political, socio-economic, and institutional factors across the agricultural supply chain (see Figure 1), through a multidimensional lens. It lays emphasis on the fact that systemic failure in attaining SDG2 is not solely due to changing weather patterns; rather, it must be seen as a compounding crisis in which socio-demographic strains collide with climate pressures (28). To commemorate this, the exponential population growth without planning, which has become a common experience in the African region, also fuels food insecurity (29). Insurgencies and terrorist onslaughts also drive food insecurity in Africa (30). Wudil et al. (31) share the view that climate change, weak governance frameworks, and entrenched corruption contribute significantly to food insecurity in the region. The distortion of rainfall patterns due to climate change and the onset of droughts entangle farmers in a web of food insecurity. This is more pronounced with farmers who are yet to embrace climate-smart agriculture (27). Engaging the discourse from a different dimension, Kurebwa and Kurebwa (32) posit that the outbreak of the COVID-19 pandemic, in no small measure, aggravated the menace of food insecurity on the continent.

The attainment of the global goals (SDGs) has remained an issue of serious concern in the 21st century (33). Wang et al. (34) assert that, in a world facing increasing environmental challenges, there is a need to pay greater attention to addressing them to facilitate the timely attainment of the global goals. (88), in their study, reveal that the adoption of agricultural technologies, embodied in climate-smart agriculture, is essential for ensuring the sustainability and resilience of the agricultural sector, especially against the vagaries of climate change. Mohammed et al. (2025), in their own study, hold the view that the nature of climate change manifestations and their associated impacts on the agricultural sector make it highly imperative to tackle them with the aid of specialized policies and high-impact technologies. Towing the same line of thought, Yan et al. (35) share the view that agricultural management has become more intensified and technologically dependent due to the effects of climate change (35). This explains the utilization of climate-smart agriculture to address the effects of climate change on the sector. From another dimension, Liu et al. (17) affirm that the switch from small-scale to large-scale agricultural production has led to intensified engagement with climate-smart agriculture.

It is a truism, validated by a myriad of literature, that climate-smart agriculture is closely related to food security. To validate this position, Akpabio et al. (36) posited that there is a nexus between climate change and agricultural sustainability. Akinsemolu et al. (37) submitted that climate-smart agriculture represents the panacea for mitigating the effects of climate change on food security. Chaurasia et al. (38) believe that it is primarily preoccupied with the role of technologies in helping agricultural practitioners mitigate their risks. Mangole et al. (39) describe it as a technique deployed to scale agricultural production. To capture the issues more succinctly, the concept of climate-smart agriculture has gained more traction in the West African sub-continent, where farming is the key profession in the sub-region (40). This is a truism in all respects, with extracts from extant literature revealing that the rate of food insecurity in the sub-region continues to surge. At the same time, the likelihood of achieving SDG 2 within the shortest possible time remains bleak. Amoak et al. (41) hold the view that the existence of a two-way causal relationship between climate change and agriculture, vis-à-vis agriculture and climate change, has made the need for climate-smart agriculture highly expedient. Zougmoré and Partey (42), in their study, allude to the fact that the engagement of climate-smart agriculture has helped, in no small measure, to curb the aftermaths of climate change on the agricultural sector. This shows that climate-smart agriculture remains one of the most potent mitigative/adaptive and climate-resilient strategies employed to enhance SDG 2 prospects globally.

*Finally, the Agricultural Innovation Diffusion Theory must be utilized to unpack why,* given the complexities of climate-smart agriculture, training has become crucial to equip farmers, especially those in rural areas, with the technical know-how required to practice it. We have also observed that climate-smart agriculture requires significant planning and governmental support, which are sometimes lacking in West Africa, explaining why the system suffers stunted growth. The unavailability of earth observation technologies to generate data, which is critical for climate-smart agriculture, equally poses a significant challenge to their adoption in West Africa (41). Although precision agricultural technologies exist in the sub-region, they are very limited and mostly visible in urban areas (43). This brings to the fore the need to critically appraise factors that enable or adversely impact climate-smart agriculture in this context. Accordingly, this study has undertaken this task by integrating these theoretical dynamics to provide a holistic and multi-layered systemic analysis.

### Assessing the state of SDG 2 in the west African sub-region

The issue of food insecurity is a global challenge, as there is barely any country in the world immune to food security challenges (44). In line with the preceding assertion, Sauer (45), in their study, provides a staggering figure of approximately 2.3 billion people who are exposed to global food insecurity. This is a clear indicator that, beyond our area of study, the issue of climate change is global in nature (46).

While food insecurity remains a global challenge, the barrage of events and factors peculiar to this clime, which have remained a recurring decimal, has made it highly susceptible to the menace compared with other sub-regions. Adepoju et al. (47) revealed that the population of food-insecure West Africans as of 2022 was pegged at over 30 million. Although the factors that promote food insecurity have been captured in this study, the sporadic rise in armed conflicts, which increases the number of refugees, has largely disrupted global food supply chains (48). This is coupled with a population explosion in the African continent, which, aside from leading to constraints on public amenities, indicates the need for massive investments in food security enhancement measures (49), as it remains a bedrock of national, economic, and social development (12).

**Figure 2:**
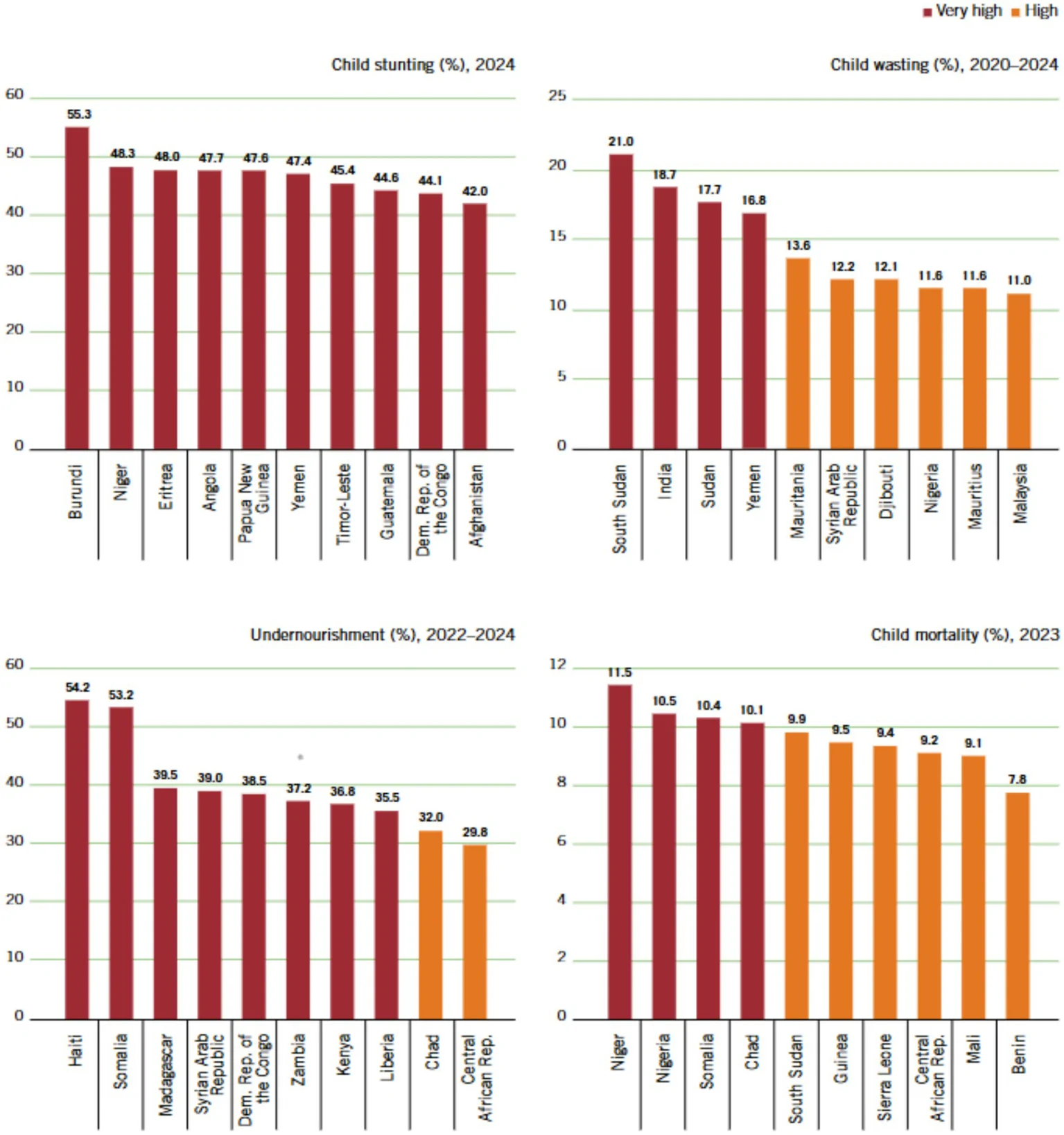
Global hunger indicators, with focus on acute hunger trends in Africa and West Africa. Reproduced from (95), licensed under CC BY-NC-ND 4.0.

Akpabio et al. (50), in their study, reveal that Africa’s food insecurity threats are more worrisome when the number of people who depend on the agricultural sector outgrows agricultural productivity. In the same vein, Sorgho et al. (51) argue that the continent’s limited adaptive capacity is a major factor contributing to the challenges it experiences in the food sector. The Sustainable Development Goals 2 (zero hunger) trajectory in West African countries can be described as far off track from the 2030 SDGs target; while global hunger index forecasts a reduction of 8.20% (673 million) by 2024, the African continent falls contrary by leaving more than 307 million (20%) of their population to face acute hunger (see Figure 2 for hunger indicators). Research has revealed that key drivers behind such contrary fall to global trend include: persistent unrest and war, increased frequency of extreme weather directly damaging crop yields, lack of income for vulnerable populations to afford nutrition and sufficient food, and systemic failures, among others (52, 53).

Building on that end, the triple threat of climate shocks, economic volatility, and violent conflict compounded a persistent cycle of stagnation across the Sahel region (52, 53). Such untoward manifestations include erratic rainfall and severe floods in 2024, which rendered Lake Chad extremely volatile, with about 236,000 individuals affected, 13,471 hectares of crops destroyed, and approximately 22,213 recorded in Cameroon and Chad, respectively (54, 55). In the same vein, climate-induced conflicts such as farmer- herder conflicts in coastal states such as Nigeria remain a central driver of food insecurity (56). Recent reports from Cadre Harmonisé (57) warn that approximately 35 million individuals are on the verge of experiencing severe hunger in 2026 (57–59).

**Figure 3:**
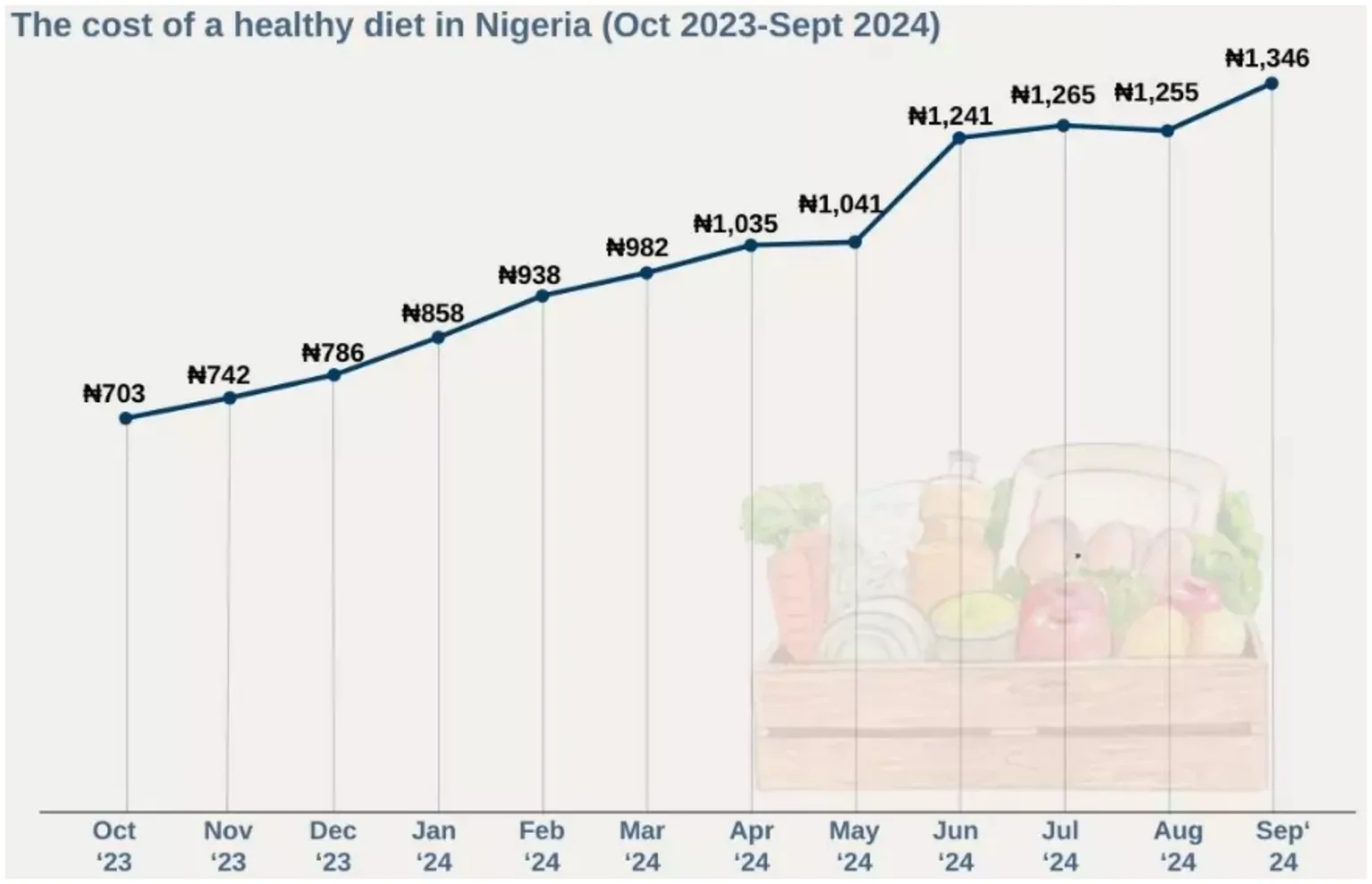
Trend in the cost of a healthy diet (CoHD) in Nigeria (October 2023–September (2024). Data is sourced from National Bureau Statistice (2023–2024).

Furthermore, the Cost of Healthy Diet (CoHD)—the minimum expense to meet nutrient needs—surged in West African states. For instance in Nigeria, from October 2023 to September 2024, there was a 91% rise with prices shifting from ₦703 to ₦1,346 (see Figure 2) (60), leaving targets 2.2 (nutrition) and 2.1 (caloric access) of SDG 2 almost impossible to reach with reasons ranging from funding gaps in agriculture R&D, slow adoption of Climate-Smart Agriculture and Indigenous Knowledge vs. Bio-technology (52). Reports estimate a US$75 billion annual financing gap for smallholder farmers across Africa (61, 62), with investment in research and development falling below the recommended 1% threshold of agricultural gross domestic product (AGDP) (91). However, despite the potential to yield results, climate-smart agriculture—an integrated approach to managing landscapes—remains low due to factors such as weak policy frameworks, expensive, inaccessible machinery, low-income smallholders, and farmers’ misconceptions of new technology (52, 58) (see Figure 3).

In light of such challenges, it is worth noting that the effect of the ECOWAS Regional Agricultural Policy (ECOWAP) on food security has been plagued by implementation delays and governance gaps within member states. Despite the key achievement of establishing Regional Food Security Reserves for regional regulations of seeds and pesticides to boost productivity and advance self-sufficiency initiatives such as Rice Offensive, the broader vision of a borderless agricultural market remain fractured due to the withdrawal of Alliances of Sahel state (Mali, Burkina Faso, and Niger), which destroys decades of trade free trade relationship under the Common External Tariff (CET) (63–65).

### Climate-smart agriculture (CSA) and SDG 2: status, problems, and prospects

Assessing the nexus between CSA and Zero Hunger (SDG 2) calls for a critical examination of regional policy frameworks such as ECOWAP, CAADP, and WAACSA, and their roles in reducing hunger and malnutrition in Western African states (52, 66). Studies show that between 1991 and 2012, ECOWAP member countries successfully reduced the prevalence of undernourishment from 24 to 11%. In a bid to achieve better results, ECOWAP adopted CSA as a core strategy to address high vulnerability to climate-related risks and to improve food security and income by incorporating CSA into farm practices, thereby mitigating greenhouse gas emissions in 2015 (67, 68). From 2015 to 2020, approximately 7 million farmers benefited from CSA in nations such as Mali, Benin, and Ghana (69, 70). Ewulo et al. (71) assert that the Climate-Smart Agriculture Investment Plan (CSAIP) is one of the pathways introduced to promote the practice of climate-smart agriculture in the sub-continent.

Furthermore, from 2020 to 2025, West African states experienced rising hunger due to climate change, which led to the reorganization of CSA priorities into five thematic areas in late 2024, through climate-resilient methods supported by regional frameworks such as the West African Initiative for Climate-Smart Agriculture (WAICSA) (66, 93). Currently, West African states are at the midpoint of transit towards Vision 2050, which prioritizes climate-smart agriculture. This prioritization holds to account that despite regional security and the withdrawal of Mali, Burkina Faso, and Niger from the bloc, ECOWAS witnessed an economic growth of approximately 4.6%, albeit alongside a high inflation rate [*Vanguard* (72)].

Other frameworks that combat this are CAADP and ECOWAP in collaboration with WAICSA, with an emphasis on the Regional Agricultural Investments Plan for Food and Nutritional Security (RAIPFNS) to achieve CSA beyond the African Union’s Malabo Declaration of 25 × 25 (feeding 25 million households) goals in 2025 (66, 73). Moreover, existing reports from studies indicate the triple-win performance measure, where adopters of CSA, such as Zimbabwe, experience a 20.2% increase in overall crop yield with a 21.15% increase in maize yield, while Kenya’s adoption of CSA technology records about a 61% increase in potato yields. Other leading adopters, such as Mali, Nigeria, Senegal, Burkina Faso, Togo, and Benin, witness a significant increase in daily calories of 217.59 kCal per capita compared with non-adopter nations (52, 69).

Compared with the conventional method with minimal climate variability adaptation, CSA adopted in the West African sub-region records yielding a superior outcome characterized by increased agricultural productivity and greater resilience to food security (90, 101). At the same time, volatile yields are more acute in Traditional systems due to factors such as significant greenhouse gas emissions, heavy losses during extreme weather events, water scarcity, and soil degradation, which frequently lead to food deficits during lean seasons (106, 115). In contrast to the foregoing, CSA practices in cereal groundnut intercropping, organic fertilizers, and soil water conservation accelerate productivity and improve the food consumption score (107). Case examination of countries such as Ghana, Mali, and Nigeria shows that CSA adoption has strong transformative capabilities for achieving SDG 2 by enhancing food security through reduced drought-related crop losses, increased calorie availability, and lower hunger rates, compared with non-adopters (109, 110). Drawing from the discourses thus far, we can infer that these realities corroborate the view that adopting CSA is not an isolated practice, as its full benefits are maximized when combined with and supported by extension services (101, 104).

Wu et al. (74), in supporting CSA, describe it as an act of innovation which can significantly benefit SDG 2; this is further supported by Nabi et al. (75) who emphasize the need to utilize smart technologies in agricultural activities. Agyekum et al. (76) hold the view that a virile CSA practice is a core requirement for the accelerated achievement of SDG 2. Hence, improving climate-smart agriculture remains a major pathway for achieving the SDGs in any climate. This position is seconded by Akpabio et al. (77), who established clear linkages between climate-smart agriculture and the attainment of SDG 2. Research has shown that, in parts of West Africa where climate-smart agriculture has been implemented, it has led to tremendous improvements in crop yields (78) and has also served as a tool for resilience in the face of climatic disruptions (79), this is quite beneficial when taking into cognizance its role in the expeditious attainment of the SDGs (35). Abegunde et al. (80) while alluding to the benefits inherent in CSA, posit that it has become more acceptable to use it to tackle SDG 2-related challenges (23).

As beneficial as climate-smart agriculture may seem, especially with the moderate degree of success recorded, it comes with significant hurdles (24, 50). To elucidate, with less than 9% of Africa’s population having access to mobile internet, engaging in climate-smart agriculture has become very challenging (81). This is further compounded by the unavailability of a robust climate information platform to support CSA, which remains a challenge for this initiative in West Africa (70). Zougmoré et al. (82) also found that small-scale farmers find it very difficult to engage in climate-smart agriculture because of the exorbitant costs associated with the practice. Hence, the relevance of Jin et al.’ (3) admonition, which emphasizes the need to pay attention to economic considerations when developing strategies to tackle environmental challenges such as climate change, especially when done with the aim of achieving sustainable development.

This study argues that most of the problems climate-smart agriculture has been plagued with are foundational and structural. Examples are conflict and displacement, climate shock (such as the submergence of 16,000 hectares of farmland in Senegal), the high cost of climate-smart tools, land tenure insecurity on the part of the farmers, top-down mismatch (CSA technological solutions are not culturally compatible with local farmers), and ECOWAS disruption (the withdrawal of Mali, Burkina Faso and Niger) creating gaps in regional co-ordination (52, 63, 83).

To address these foundational and structural challenges for the Agenda 2050, swift transition, blended finance initiatives such as the West African Initiative for Climate-Smart Agriculture (WAICSA)—embedded in ECOWAS’s Regional Agriculture Development Fund (ECOWADF) financial structure for smallholders. WAICSA, in collaboration with food and agriculture organizations such as AICCRA/CGIAR, should invest in projects that advance climate-resilient methods, early warning systems, insurance pilots, and diversified farming to buffer against climate shocks. To cushion the impact of ECOWAS disruption on advancing food security through CSA, the AES-ECOWAS dialogue and depoliticized co-operation could de-escalate tensions, with incentives for co-operation on food security. A bottom-up participatory approach should be used to address the top-down mismatch, strengthening stakeholder capacity locally and internationally to achieve balance and cultural compatibility.

### Recommendations

While advancing progress toward SDG 2 (Zero Hunger) through climate-smart agriculture to meet the 2030 target, in alignment with the region’s long-term Vision 2050, the study recommends depoliticized and pragmatic cooperation between ECOWAS and AES for food security; accelerating and scaling up WAICSA as the flagship blended-finance mechanism for CSA; promoting bottom-up, participatory, and culturally compatible CSA adoption; increasing public and private investment in agriculture R&D, infrastructure, and nutrition; strengthening monitoring systems, early warning mechanisms, and multi-sectoral coordination.

First, to move beyond the effect of ECOWAS disruption and AES, there is a need to foster functional collaboration on food security to mitigate the fragmentation impact. This collaboration should address specific issues via neutral platforms such as Comité permanent inter-États de lutte contre la sécheresse au Sahel (CILSS), Réseau de prévention des crises alimentaires (RPCA), and AU-led forums to access shared mechanisms such as ECOWAS Regional Food Security Reserve, cross-border agricultural input protocols such as seeds and pesticides, early warning systems via Cadre Harmonisé and CILSS, and joint humanitarian corridors. Moreover, accessibility to incentives such as shared insurance pilots, joint humanitarian and technical exchanges on CSA to mitigate rising food prices and worsening hunger in West African states.

Second, to accelerate and scale WAICSA as the flagship blended finance mechanism for CSA. WAICSA as embedded in the ECOWAS Regional Agriculture Development Fund (ECOWADF) and managed ECOWAS Bank for Investment and Development (EBID) for financing and the Regional Agency for Agriculture and Food (RAAF) for technical assistance should mobilize substantial resources and build meaningful partnerships, such as the one they did with Global Green Growth Institute in addressing agricultural input needs across West African states through subsidized loans, grants and diversified farm systems among others. Moreover, they should prioritize closing the US$75 billion annual financing gap for smallholders with the desire to reach at least 90,000 households and convert 185,000 hectares to CSA practices in pilot phases (initially targeting 6 ECOWAS states, with replication across all 15).

Third, promoting the adoption of CSA from a bottom-up, participatory, and culturally compatible perspective. Integrating local knowledge with proven practices such as conservation agriculture, agroforestry, rainwater harvesting, better livestock management, drought-resistant varieties, and gender-responsive fertilization through farmer-led demonstration farms, extension services, affordable machinery leasing, and gender-responsive training for women, young people, and pastoralist groups will be more successful and achieve the CSA’s primary objective in West Africa.

Fourth, leveraging public and private investment in agriculture R&D, infrastructure, and nutritional capacity will be more beneficial. ECOWAS member states and national governments should meet, discuss, and set a new target of commitment that is development-push and more realistic than the CAADP/Malabo allocation of 1% of agricultural GDP to R&D and extension, while attracting blended finance and private-sector participation in CSA value chains. Investments in rural irrigation, storage, roads, and regional self-sufficiency initiatives to reverse Nigeria’s 91% surge in healthy diet costs and target SDG 2.1 (caloric access) and 2.2 (nutrition) and other African Countries.

Finally, to strengthen monitoring, early warning and multisectoral convergence, the Cadre Harmonisation and IPC systems for early forecasting, the integration of CSA priorities into the national disaster risk frameworks and the ECOWAP and AfDB plans, coupled with mobilization of funds to monitor progress, should be strengthened through participatory reviews and through partnership and engagement of civil society and institutions like FAO, WFP, CGIAR and the AfDB.

## Conclusion

West Africa divergence from global hunger reduction forecasts was 20% in 2024, while approximately 42 million people in 2025 face acute hunger due to intertwined threats (the triple threat of climate change, conflict, and economic volatility), ECOWAS disruptions, conflict and displacement, climate shock, the high cost of climate-smart tools, land tenure insecurity, top-down mismatch which create systemic gaps making SDG 2 targets by 2030 increasingly out of reach without bold action. However, amid these challenges, ECOWAS recorded economic growth of approximately 4.6% in 2025, despite a high inflation rate, with projections of 5% in 2026, while CSA initiatives have delivered tangible gains in yields, calories, and farmer resilience across countries such as Mali, Ghana, and Benin. The study’s recommendation of depoliticized, pragmatic cooperation between ECOWAS and AES for food security, accelerating and scaling WAICSA as the flagship blended finance mechanism for CSA, promotion of bottom-up, participatory, and culturally compatible CSA adoption, fostering of public and private investment in agriculture R&D, infrastructure, and nutrition, strengthening of monitoring, early warning, and multi-sectoral alignment can avert deepening crises, achieve the triple-win of productivity, adaptation, and mitigation, and build equitable, resilient agrifood systems that deliver zero hunger and sustainable prosperity for generations to come in West African states. It is evident that issues with climate change top the chart among the leading constraints facing the agricultural sector in the region (84). However, despite the limitations that come with this experience, designing a mitigative strategy to surmount this challenge remains the solution to containing its untoward impacts on SDG 2. In a nutshell, while we hold the view that long-term sustainability is the major driver of technological adoption as encapsulated in CSA (85), it is important to state that, irrespective of the technology engaged to support any sustainable development goal, complex decision-making must be carried out (33). This brings to the fore our persistent calls for a revamping of CSA initiatives and a beefing up of its policies for the expeditious attainment of SDG 2.
